# Relaxant Effect of Essential Oil of *Artemisia herba-alba* Asso. on Rodent Jejunum Contractions

**DOI:** 10.3797/scipharm.1106-13

**Published:** 2012-01-12

**Authors:** Mohammed Aziz, Ahmed Karim, El Mokhtar El Ouariachi, Abdelhamid Bouyanzer, Souliman Amrani, Hassane Mekhfi, Abderrahim Ziyyat, Ahmed Melhaoui, Mohamed Bnouham, Abdelkhaleq Legssyer

**Affiliations:** 1 Laboratoire de Physiologie et d’Ethnopharmacologie, Université Mohammed I, Faculté des Sciences, BP 717, 60000, Oujda, Morocco; 2 Laboratoire de Chimie appliquée et Environnement, Université Mohammed I, Faculté des Sciences, BP 717, 60000, Oujda, Morocco; 3 Laboratoire de Biochimie, Université Mohammed I, Faculté des Sciences, BP 717, 60000, Oujda, Morocco; 4 Laboratoire de Chimie Organique, Macromoléculaire et Produits Naturels, Université Mohammed I, Faculté des Sciences, BP 717, 60000, Oujda, Morocco

**Keywords:** *Artemisia herba-alba* Asso., Essential oil, Rodent jejunum, Smooth muscle

## Abstract

*Artemisia herba-alba* Asso. is a shrub commonly encountered in Morocco. It is used in traditional medicine for treating intestinal disorders. The essential oil extracted from the plant’s aerial parts reversibly relaxed the spontaneous tonus of the rabbit jejunum in a reversible concentration dependent manner with an IC_50_ value of 97.33 ± 2.59 ng/ml and reversed the tonic contraction of rat jejunum induced by 75 mM KCl and 10^−6^ M carbachol with IC_50_ values of 115.5 ± 3.05 and 119.4 ± 20.86 ng/ml, respectively. The pre-treatment of the latter isolated intestine with this essential oil produced a dose-dependent shift of the Ca^++^ and CCh dose-response curve to the right, with suppression of the maximal effect, similar to the non-competitive antagonist effect on muscarinic receptors and calcium channel, respectively.

## Introduction

The genus *Artemisia*, included in the family Asteraceae, comprises a variable number of species (from 200 to over 400, depending on the authors) found throughout the northern half of the world [1]. *Artemisia herba-alba* Asso., a medicinal and aromatic dwarf shrub, is commonly grown in Mediterranean basin [2]. In Morocco, this plant grows in very vast steppes. It is used extensively in traditional medicine to treat helminthiasis, diabetes mellitus and other conditions such as jaundice [3]. Also, the antihyperglycaemic [4], antimicrobial [5], antioxidant, antispasmodic, anti-venom, nematicidal, anthelmintic, anti-leishmanial, neurological, pesticidal and antibiotic resistant inhibitor activities of this plant have previously been reported [6]. Moreover, the aqueous extract significantly increased gastrointestinal transit time and the reaction time to thermal stimuli [7]. Furthermore, the species of this genus are widely used in the pharmaceutics, cosmetics and food industry. *Artemisia caerulescens* has analgesic, antipyretic and anti-inflammatory actions [8]. *Artemisia capillaris* dilates blood vessels [9], and other species of *Artemisia* have antimicrobial and antimalarial activities [10, 11].

Medicinal plants in Morocco [12–14] and also in the world [15, 16] are widely used in the treatment of digestive disorders. In this research we have studied the effect of the aerial-part essential oil of *Artemisia herba-alba* Asso. (EOAH) on contraction of isolated rabbit and rat jejunums *in vitro* to examine their potential relaxant activity.

## Results and Discussion

The analysis of essential oil obtained from the aerial part of *Artemisia herba-alba* Asso. revealed the presence of 34 identified compounds contributing to 86.2% of the oil. The essential oil contains as main constituents: 30.6 % (w/v) of chrysanthenone, 24.4% of camphor, 4.5 % of camphene, 4.3 % of filifolone, 3 % of 1,8-cineol, 2.7 % of β-thujone, 2.4 of α-thujone and 2 % of α-pinene [17]. It has been reported that the chemical composition of EOAH found abundantly in the Mediterranean basin varies according to the genetic characteristics [5] and climatic, seasonal, geographical and geological differences where the plant is collected. Analysis of oil samples from Morocco has established the existence of at least seven chemotypes of the so-called armoise oil [18–20]. Other different chemotypes were found in oil samples from Mediterranean basin countries. [2, 21–24]. Phytochemical investigations have proven that this genus is rich in sesquiterpenes and monoterpenes [25, 26].

Because of its traditional use as an antispasmodic remedy, the EOAH was tested for its possible spasmolytic effect on isolated rabbit and rat jejunum preparations.

The isolated rabbit jejunum preparations had spontaneous contractions when they were mounted in the tissue bath under 1 g tension. Exposure of the preparations from 0 to 200 ng/ml of the essential oil reduced the average amplitude of the spontaneous contractions to 100 % of control with an IC_50_ value of 97.33 ± 2.59 ng/ml. This effect was reversible as the tissue regained its spontaneous activity after washing the tissue a couple times with the fresh bathing fluid. Verapamil used as a reference drug showed a total inhibition at 1 μM.

**Fig. 1.**
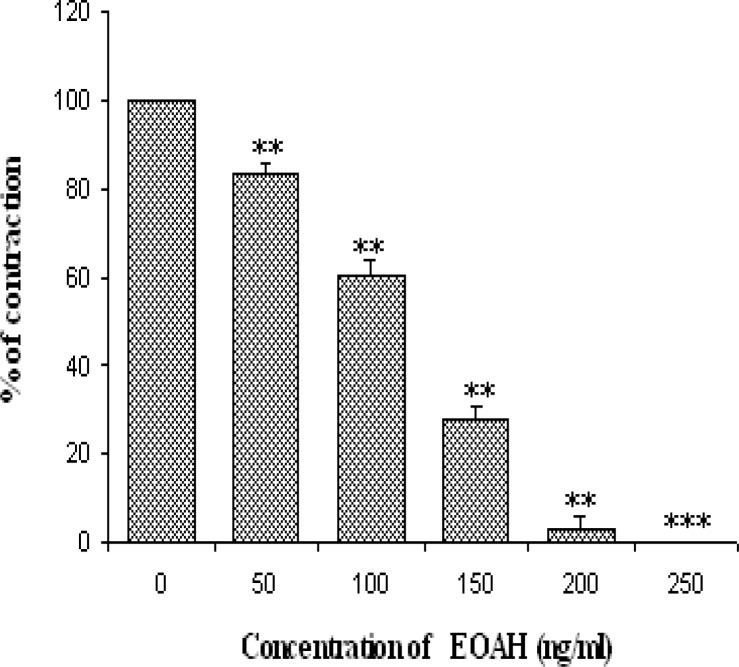
Relaxant effect of essential oil of *Artemisia herba-alba* on K^+^ (75mM) induced contractions.

**Fig. 2.**
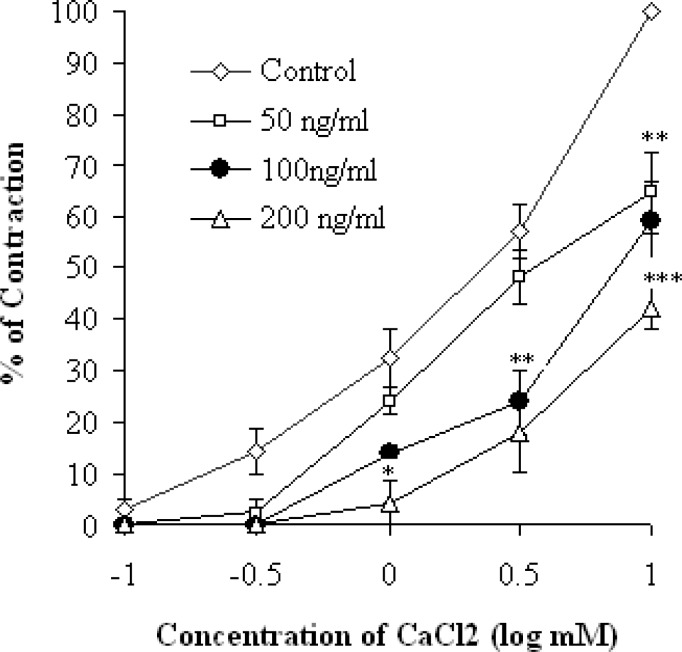
Cumulative log concentration-response curves (±S.E.M, Student’s t-test; n = 6) for CaCl_2_ in the presence and absence of the essential oil of *Artemisia herba-alba.*

The contraction of smooth muscle is dependent on an increase in the concentration of cytosolic Ca^++^. The latter may come either from the extracellular medium or from the sarcoplasmic reticulum [27]. In the case of the increase in the concentration of K^+^ above 30 mM in the extracellular medium, a depolarization of the membrane occurs, and consequently the voltage-operated Ca^++^ channels (VOCCs) open and make Ca^++^ penetrate into cytoplasm [28]. Agents that inhibit contraction induced by KCl should somehow inhibit the entry of Ca^++^ ions or otherwise inhibit the intercellular contraction mechanism [29]. To confirm the interaction of EOAH with voltage dependent Ca^++^ channel, the tissue was pre-treated with high potassium (75mM). This latter produced a sustained tonic contraction, which was maintained during the course of experiments. EOAH in a concentration-dependent manner (50–200 ng/ml) inhibited the rat jejunum contractions induced by 75 mM KCl with an IC_50_ value of 115.5 ± 3.05 ng/ml. With 200 ng/ml bath concentration EOAH abolished the response to KCl. (Figure 1). Therefore, inhibition of the contraction of rat jejunum by the EOAH reflects the limited entry of Ca^++^ through VOCCs. It is quite possible that one or more components of EOAH block these channels. This hypothesis was strengthened when pre-treatment of the tissue with the plant essential oil caused a concentration dependent rightward shift in the concentration-response curves of CaCl_2_ (Figure 2). Furthermore, Verapamil also reduced the maximal response in curves induced by CaCl_2_ (Table 1). These Organic calcium channel antagonists inhibit more markedly the entry of calcium through the L-VOCCs.

**Fig. 3.**
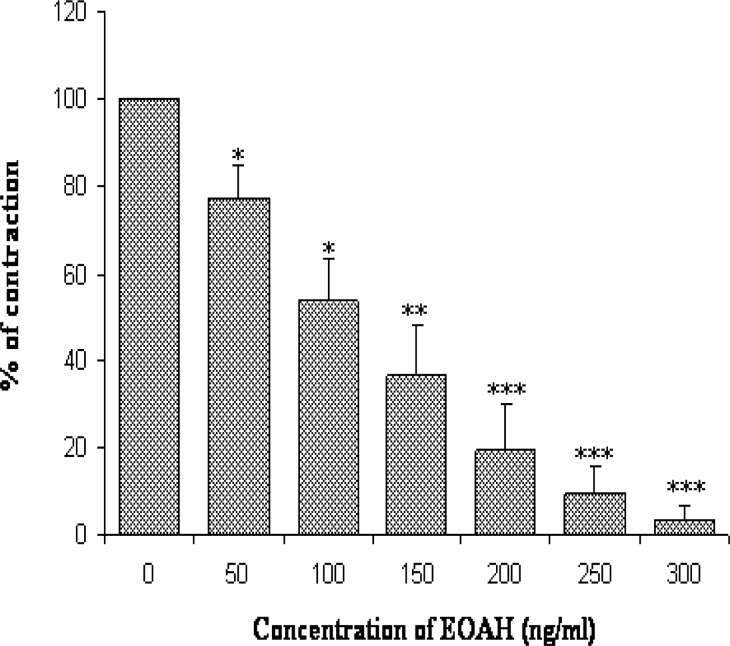
Relaxant effect of essential oil of *Artemisia herba-alba* on Carbachol (10^−6^ M) induced contractions.

**Fig. 4.**
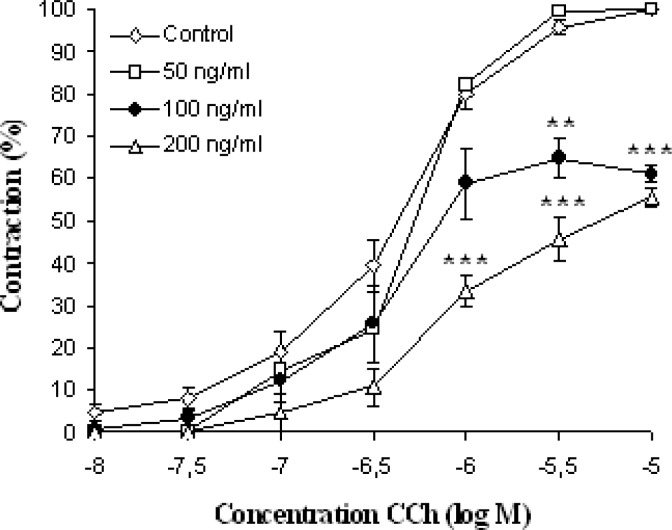
Cumulative log concentration-response curves (±S.E.M, Student’s t-test; n = 6) for CCh in the presence and absence of the essential oil of *Artemisia herba-alba*.

Carbachol produced sub-maximal contractions at 10^−6^ M. EOAH in a concentration-dependent manner (50–300 ng/ml) inhibited the rat jejunum contractions induced by CCh with an IC_50_ value of 119.4 ± 20.86 ng/ml (Figure 3). Similar effects were obtained with papaverine, a non specific smooth muscle relaxant, on the dose response curves induced by CCh (Table 1). Carbachol, a cholinomemetic drug, interacts with muscarinic receptors on intestinal smooth muscle cell membrane [30]. The main muscarinic cholinergic receptor subtypes in the gastrointestinal tract are M2 and M3. Both are coupled to G-proteins but exert different intracellular effects. M2 acts predominantly via inhibition of the enzyme adenylate cyclase and, hence, decreases the levels of cAMP in the cell while M3 causes activation of phospholipase C and subsequent hydrolysis of phosphatidyl inositol biphosphate (PIP2) into inositol triphosphate (IP3) and diacylglycerol (DAG) [31]. The IP3 interacts with reticulum receptor and releases the stored reliculum calcium in the cytoplasm. Non-competitive antagonists of muscarinic receptors antagonised the response to Ach by antagonising the muscarinic receptors and, therefore, with altering the maximum response they shift Ach concentration–response curve to the right [32]. The same result was obtained with our oil (Figure 4). We can propose the hypothesis that a component (or more) of EOAH exerts a non-competitive antagonist effect on muscarinic receptors.

Inhibition of both CCh and CaCl_2_-induced contraction may indicate that the spasmolytic compound included in the EOAH is not a specific receptor antagonist.

Furthermore, since we deal directly with the total essential oil, there may be more than one relaxant effect compound involved. As components of EOAH are hydrophobic, they can easily cross the membrane and act directly on the internal pathways involved in the relaxation of smooth muscle cells.

One or more components of EOAH could act on the plasma membrane, the muscarinic receptors, the VOCCs or one step of intracellular pathways that contribute to the contraction of smooth muscle cells. This spasmolytic activity of EOAH may be due to the presence of camphor, terpinene [33], 1,8-cineol [34], α- and β-pinene [35], which have been reported to be smooth muscle relaxants. However, the presence of other spasmolytic compound(s) cannot be excluded.

In Morocco, Artemisia is mainly used as an infusion. It would be interesting to know the amount of essential oils contained in this infusion. To our knowledge there is no published article on this subject. There most likely would be less essential oils than the amount directly extracted from the plant, as this has been the case for other plants. For example, comparison of the total essential oil of *Rosmarinus officinalis* yield quantified by hydrodistillation of the infusion (0.36% v/w) with the essential oil yield of the leaves (1.84% v/w) revealed that only 19.6% of the initial oil could be extracted by infusion [36]. This study showed remarkable differences between the relative proportions of chemical classes in the two types of extraction of rosemary isolated essential oils.

In conclusion, this study showed that *Artemisia herba-alba* essential oil possesses a significant inhibition effect on jejunum contractions. It is possible that other hydrophilic constituents in the *Artemisia herba-alba* infusion can relax the jejunum. Therefore, it seems to be a useful herbal medicine for the treatment of gastrointestinal spasms. However, further studies need to investigate its potential effects on other smooth muscle and any undesirable effects on other organs.

## Experimental

### Solutions and Drugs

The solutions used had the following composition:

Normal Krebs-Henseleit Buffer (KHB) solution composed of (mM) NaCl, 118; KCl, 4.7; CaCl_2_, 2.5; MgSO_4_, 1.2; NaHCO_3_, 25; KH_2_PO_4_, 1.2 and glucose 10.
High K^+^ KHB (75mM); NaCl, 48; KCl, 75; CaCl_2_, 2.5; MgSO_4_, 1.2; NaHCO_3_, 25; KH_2_PO_4_, 1.2 and glucose 10.
Calcium-free high K^+^; KHB (75mM); NaCl, 48; KCl, 75; CaCl_2_, 0.0; MgSO_4_, 1.2; NaHCO_3_, 25; KH_2_PO_4_, 1.2 and glucose 10.
Calcium-free KHB; NaCl, 121.7; KCl, 4.7; CaCl_2_, 0.0; MgSO_4_, 1.2; NaHCO_3_, 25; KH_2_PO_4_, 1.2 and glucose 10.

All these solutions were made up in distilled water, and the pH was adjusted to 7.4.

The following drugs were used for the experiments: carbamylcholine chloride (carbachol, CCh) and verapamil hydrochloride were purchased from Sigma, papaverine hydrochloride from Fluka and dimethyl sulfoxide (DMSO) was purchased from Prolabo.

### Plant material

The fresh plant of *Artemisia herba-alba* Asso (Asteraceae) was collected locally during the flowering time (in May 2008) from North east area of Morocco; the botanical identification was done by Professor B. Haloui at the department of Biology, Faculty of Sciences, University Mohammed the First, Oujda, Morocco. A voucher specimen (N° 43130) was previously deposited in Scientific Institute of Rabat.

### Preparation and analysis of essential oil

The dried aerial parts of *Artemisia herba-alba* were submitted to hydrodistillation for 4 h using a Clevenger-type apparatus. *Gas chromatographic* analyses were carried out using a Perkin Elmer Clarus 600 fast GC apparatus equipped with a single injector and two flame ionization detectors (FID). Sample oil was analyzed with a Perkin Elmer TurboMass detector, directly coupled to a Perkin Elmer Autosystem XL. The essential oil of *Artemisia herba-alba* (AHEO) contains as main constituents: 30.6 % (w/v) of chrysanthenone, 24.4% of camphor, 4.5 % of camphene, 4.3 % of filifolone, 3 % of 1,8-cineol, 2.7 % of β-thujone, 2.4 of α-thujone and 2 % of α-pinene [17].

### Animals

New-Zealand rabbits (2–2.5 kg) and Wistar rats (200–250 g), maintained under standard condition at the animal house of the Department of Biology, Mohammed First University, Oujda, Morocco, and fed with standard diet with water ad libitum were used for the experiment. Animals were fasted overnight and had access to water ad libitum prior to experimentation. All procedures concerning animals were carried out in an ethical manner by following guidelines as set by the World Health Organization and conforming to the European Community guiding principles in the care and use of animals (86/609/CEE, CE Off J No. L358, 18 December 1986). Under these experimental conditions, the rat jejunum behaves as a quiescent smooth muscle preparation and is considered more useful for studying the contractile responses of agonists like carbachol or KCl rich medium, whereas, rabbit jejunum exhibits spontaneous rhythmic contractions, allowing the relaxant (spasmolytic) activity to be tested directly without the use of an agonist.

### Spasmolytic study

A portion of rat and rabbit jejunums (2 cm) was removed and mounted in 10 ml organ baths containing Krebs-Henseleit buffer (KHB). The bath solution was maintained at 37°C, pH 7.4 and gassed continuously with air bubbling. A 60 min equilibration period was allowed during which the physiological solution was changed every 15 min. EOAH was dissolved with the vehicle DMSO (1%) and added to the organ bath.

### Effect of EOAH on spontaneous contractions of isolated rabbit jejunum

The spasmolytic activity of the plant material was studied by using isolated rabbit jejunum preparations, the segments were suspended to the organ bath containing KHB solution. After stabilization of smooth muscle spontaneous contractions of rabbit jejunum, the cumulative doses of EOAH (50–200 ng/ml) were added to the organ bath.

### Relaxant effect on K^+^ and CCh induced contractions of rat jejunum

The rat jejunum was contracted with K^+^ (KCl 75 mM) or carbachol (CCh, 10^−6^M) to a maintained tone. At this point the essential oil was added to the bath.

### Inhibition of dose-response to Carbachol

Cumulative dose-response curves for carbachol (CCh) were obtained for the tissues according to the method of Van Rossum [37]. After a stabilization period of 60 min, CCh (10^−8^−10^−5^ M) was added to the organ bath, and different doses of the EOAH were added to the bath 5 min before commencing the dose response curve of the agonist.

### Inhibition of dose-response to CaCl_2_

After an initial incubation period of 60 min in normal KHB’s solution, the nutrient solution was replaced by calcium-free KHB during 15 min, then replaced by calcium-free hyperpotassic medium (K^+^ 75 mM). Cumulative dose-response curves to CaCl_2_ (0.1, 0.3, 1, 3, 10 mM) were obtained in the presence of different doses of essential oil.

### Statistics

The results are expressed as means ± S.E.M. The statistical significance of data was analyzed using Student’s t–test, *P*<0.05 was considered as significant. The 50% inhibitory concentration (IC_50_) was determined by linear regression method.

## Figures and Tables

**Tab. 1.** EC_50_ and maximum effect values obtained from the cumulative dose-response curves to CCh and CaCl_2_ in rat jejunum in the presence and absence of the essential oil of *Artemisia herba-alba*.

**Antagonist**	**CCh**		**CaCl_2_**	

	**EC_50_ (M)**	**E_max_ ± S.E.M**	**EC_50_ (M)**	**E_max_ ± S.E.M**
Control	2.81×10^−6^	100	4.27×10^−3^	100
EOAH (ng/ml)				
50	1.99×10^−6^	100 ± 5.32	6.55×10^−3^	64.5 ± 18.8
100	1.66×10^−5^	59.1 ± 7.72	8.16×10^−3^	59.1 ± 7.72
200	2.64×10^−5^	42.3 ± 4.35*	1.15×10^−2^	42.3 ± 4.35*
Papaverine (10^−5^ M)	3.68×10^−5^	32.8 ± 2.06**	–	–
Verapamil (10^−6^ M)	–	–	1.13×10^−2^	46.6 ± 4.2**

Number of experiments n = 6,

* *P* < 0.05,

** *P* < 0.01 statistically significant difference from control

## References

[1] Marco JA, Barbera O, Atta-ur-Rahman (1990). Natural products from the genus Artemisia L. Stud Nat Prod Chem.

[2] Vernin G, Merad O, Vernin GMF, Zamkotsian RM, Parkanyi C, Charalambous G (1995). GC–MS analysis of *Artemisia herba-alba* Asso essential oils from Algeria. Food Flavors: Generation, Analysis and Process Influence.

[3] Migahid AM (1978). Flora of Saudi Arabia.

[4] AI-Khazarji SM, AI-Shamony LA, Twaij HAA (1993). Hypoglycaemic effect of. Artemisia herba-alba.

[5] Imelouane B, El Bachiri A, Ankit M, Khedid K, Wathelet JP, Amhamdi H (2010). Essential oil composition and antimicrobial activity of *Artemisia herba-alba* Asso grown in Morocco. Banats J Biotechnol.

[6] Mohamed AE-HH, El-Sayed MA, Hegazy ME, Helaly SE, Esmail AM, Mohamed NS (2010). Chemical constituents and biological activities of *Artemisia herba-alba*. Rec Nat Prod.

[7] Husnia Marrif I, Ali BH, Hassan KM (1995). Some pharmacological studies on *Artemisia herba-alba* (Asso.) in rabbits and mice. J Ethnopharmacol.

[8] Moran A, Martin ML, Montero MJ, Ortiz-de-Urbina AV, Sevilla MA, San Roman L (1989). Analgesic, antipyretic and anti-inflammatory activity of the essential oil of *Artemisia caerulescens* subsp, gallica. J Ethnopharmacol.

[9] Yamahara J, Kobayashi G, Matsuda H, Katayama T, Fujimura H (1989). Vascular dilatory action of *Artemisia cappilaris* bud extracts and their active constituent. J Ethnopharmacol.

[10] Kaur S, Shinna GK (1982). Antibacterial activity of volatile oils and their important constitutents from some indigenous plants. Indian J Phys Nat Sci.

[11] Yang Q, Shi W, Li R, Gan J (1982). The antimalarial and toxic effect of Artemisia in animal models. J Tradit Chin Med.

[12] Bellakhdar J (1997). La pharmacopée marocaine traditionnelle. Médecine arabe ancienne et savoirs populaires.

[13] Aziz M, Tab N, Karim A, Mekhfi H, Bnouham M, Ziyyat A, Melhaoui A, Legssyer A (2006). Relaxant effect of aqueous extract of *Cistus ladaniferus* on rodent intestinal contractions. Fitoterapia.

[14] Karim A, Berrabah M, Mekhfi H, Ziyyat A, Legssyer A, Bouali A, Haloui B, Amrani S, Aziz M (2010). Effect of essential oil of *Anthemis mauritiana* Maire & Sennen flowers on intestinal smooth muscle contractility. J Smooth Muscle Res.

[15] Cortés AR, Delgadillo AJ, Hurtado M, Domínguez-Ramirez AM, Medina JR, Auki K (2006). The Antispasmodic Activity of *Buddleja scordioides* and *Buddleja perfoliata* on Isolated Intestinal Preparations. Biol Pharm Bull.

[16] Emendörfer F, Bellato F, Noldin VF, Cechinel-Filho V, Yunes RA, Monache FD, Cardozo AM (2005). Antispasmodic Activity of Fractions and Cynaropicrin from *Cynara scolymus* on Guinea-Pig Ileum. Biol Pharm Bull.

[17] Ouachikh O, Bouyanzer A, Bouklah M, Desjobert JM, Costa J, Hammouti B (2009). Application of Essential Oil of *Artemisia herba-alba* as green corrosion inhibitor for steel in 0.5M H2SO4. Surf Rev Letters.

[18] Benjilali B, Sarris J, Richard H (1982). Nouveaux chémotypes d’*Artemisia herba-alba*. Sci Aliment.

[19] Ouyahya A, Negre R, Viano J, Lozano YF, Gaydou EM (1990). Essential oils from Moroccan *Artemisia negrei, A. mesatlantica* and *A. herba-alba*. Lebensm Wiss Technol.

[20] Lawrence BM (1993). Armoise oil. Natural Flavor and Fragrance Materials. Perfumer and Flavorist (Ed.), Essential Oils 1988–1991.

[21] Segal R, Feuerstein I, Danin A (1987). Chemotypes of *Artemisia herba-alba* in Israel based on their sesquiterpene lactone and essential oil constitution. Biochem Syst Ecol.

[22] Yashphe J, Feuerstein I, Barel S, Segal R (1987). The antibacterial and antispasmodic activity of *Artemisia herba-alba* Asso. II. Examination of essential oils from various chemotypes. Int J Crude Drug Res.

[23] El-Sayed AM, Seida AA (1990). Comparative study of the major constituents of the essential oils of wild and cultivated Egyptian *Artemisia herba-alba* with those of plants produced abroad. Bull Fac Pharm.

[24] Salido S, Valenzuela LR, Altarejos J, Nogueras M, Sánchez A, Cano E (2004). Composition and infraspecific variability of *Artemisia herba-alba* from Southern Spain. Biochem Syst Ecol.

[25] Ahmed AA, Abou El-Ela M, Jakupovic J, Seif El-Din AA, Sabri N (1990). Eudesmanolides and other constituents from *Atemisia herba-alba*. Phytochemistry.

[26] Tang HQ, Hu J, Yang L, Tan RX (2000). Terpenoids and flavonoids from Artemisia species. Planta Med.

[27] Karaki H, Weiss GB (1988). Calcium release in smooth muscle. Life Sci.

[28] Bolton TB (1979). Mechanism of action of transmitters and other substances on smooth muscles. Physiol Rev.

[29] Godfraind T, Miller R, Wibo M (1986). Calcium antagonism and calcium entry blockade. Pharmacol Rev.

[30] Goyal RK (1988). Identification, localization and classification of muscarinic receptor subtypes in the gut. Life Sci.

[31] Ehlert FJ, Thomas EA, Gerstin EH, Griffin MT, Eglen RM (1997). Muscarinic receptors and gastrointestinal smooth muscle. Muscarinic Receptor Subtypes in Smooth Muscle.

[32] Hajhashemi V, Sadraei H, Ghannadi AR, Mohseni M (2000). Antispasmodic and anti-diarrhoeal effect of *Satureja hortensis* L. essential oil. J Ehnopharmacol.

[33] Astudillo A, Hong E, Bye R, Navarette A (2004). Antispasmodic activity of extracts and compounds of *Acalypha phleoides* Cav. Phytother Res.

[34] Madeira SVF, Rabelo M, Soares PMG, Souza EP, Meireles AVP, Montenegro C (2005). Temporal variation in chemical composition and relaxant action of the essential oil of *Ocimum gratissimum* L. (Labiatae) on guinea-pig ileum. Phytomedicine.

[35] Sadraei H, Asghari GR, Hajhashemi V, Kolagar A, Ebrahimi M (2001). Spasmolytic activity of essential oil and various extracts of *Ferula gummosa* Boiss. on ileum contractions. Phytomedicine.

[36] Tschiggerl C, Bucar F (2010). Investigation of the Volatile Fraction of Rosemary Infusion Extracts. Sci Pharm.

[37] Van Rossum JM (1963). Cumulative dose-response curves: II. Technique for the making of dose-response curves on isolated organ and evaluation of drug parameters. Arch Int Pharmacolodyn Ther.

